# Supplementary material for: The value of intra-operative electrographic biomarkers for tailoring during epilepsy surgery: from group-level to patient-level analysis
Source: Sci Rep. 2020 Sep 4;10:14654. doi: 10.1038/s41598-020-71359-2 (PMC7474097; doi:10.1038/s41598-020-71359-2)
Supplement: Supplementary file 1 — Supplementary Information. [file 41598_2020_71359_MOESM1_ESM.docx]

The value of intra-operative electrographic biomarkers for tailoring during epilepsy surgery: from group-level to patient-level analysis.

Matteo Demuru^1,2^, Stiliyan Kalitzin^1,3^, Willemiek Zweiphenning^2^, Dorien van Blooijs^2^, Maryse van ’t Klooster^2^, Pieter Van Eijsden^2^, Frans [Leijten](https://www.sciencedirect.com/science/article/abs/pii/S1935861X18309914?via%3Dihub#!)^2^, Maeike Zijlmans^1,2^, RESPect group

1 *Stichting Epilepsie Instellingen Nederland (SEIN), The Netherlands*

2 *Brain Center Rudolf Magnus, Dept. of Neurology & Neurosurgery, University Medical Center Utrecht, The Netherlands*

3 *Image Sciences Institute, University Medical Center Utrecht, The Netherlands*

Supplementary materials

**Classical frequency bands (PLV and PLI)**

For PLV and PLI we additionally investigated the functional connectivity in different frequency bands (using a bipolar montage). Different studies reported an effect on the classical frequency bands,especially results in theta band have been reported[^1–3^](https://paperpile.com/c/UFn3f7/SWCM+DXsn+tOpx). [Figure S1](#_zfebsfz8om0a) shows the overall comparison between the distribution of all post-resected biomarker values (in cured patients) and the pre-resected resected biomarker values across all patients. We could partly corroborate previous results: PLI was significant (p<0.01) in delta, theta and alpha band; while for PLV we found a significant effect only in alpha band.

[Figure S2](#_mjkxdkesmfpz) shows the comparison between the distribution of maximum values computed in pre-resection resected channels for improved patients and maximum post-resection channel values for cured patients. Each coloured dot represents the maximum value of the biomarker for each individual patient across all the channels and situations. None of the biomarkers in any frequency band was found significant (alpha-level 0.01). Furthermore, for PLI, which was one of the selected biomarkers used in analysis for definition of the reference threshold, we observed that the threshold selected in the gamma band (reported in Figure 3 on the main manuscript) was lower than any of the thresholds computed in the remaining frequency bands (i.e. for each frequency band, the maximum across all channels and all patients in the post-resection cured group). Therefore, for PLI choosing a unique threshold as the maximum across the frequency bands will decrease the overall performance (i.e. number of patients for which at least one biomarker value is higher than the reference threshold).


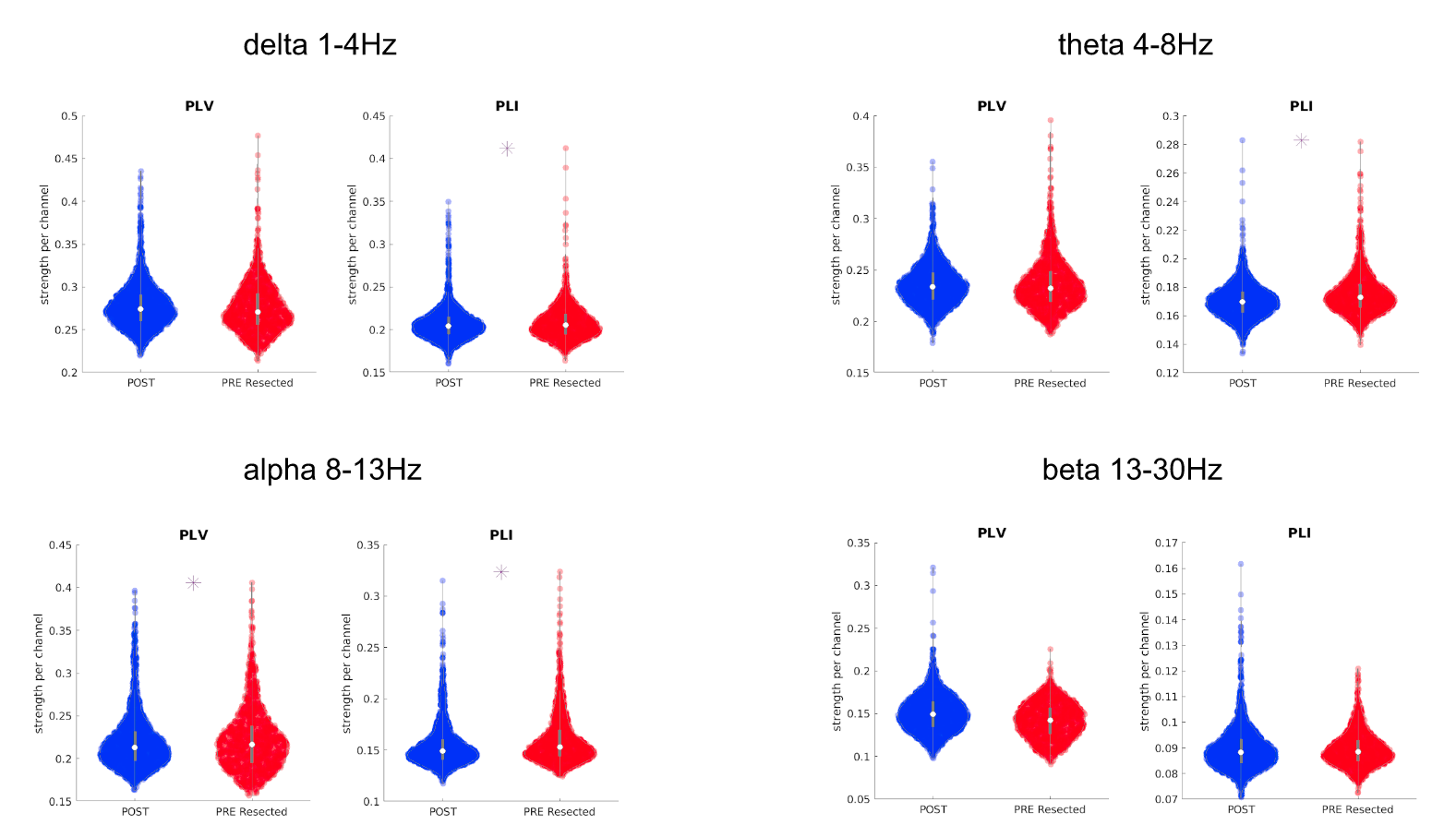


### Figure S1 Comparison between biomarker distributions values computed on pre-resection resected channels (red) and post-resection channels (blue) in cured patients using a bipolar montage. The presence of an asterisk indicates that the two distributions are significantly different (p < 0.01 one-sided Kolmogorov-Smirnov test). The y-axis for the biomarkers represents the strength. Inside each violin-plot a boxplot is depicted in gray with the median value highlighted with a white dot. PLV = Phase Locking Value; PLI = Phase Lag Index.


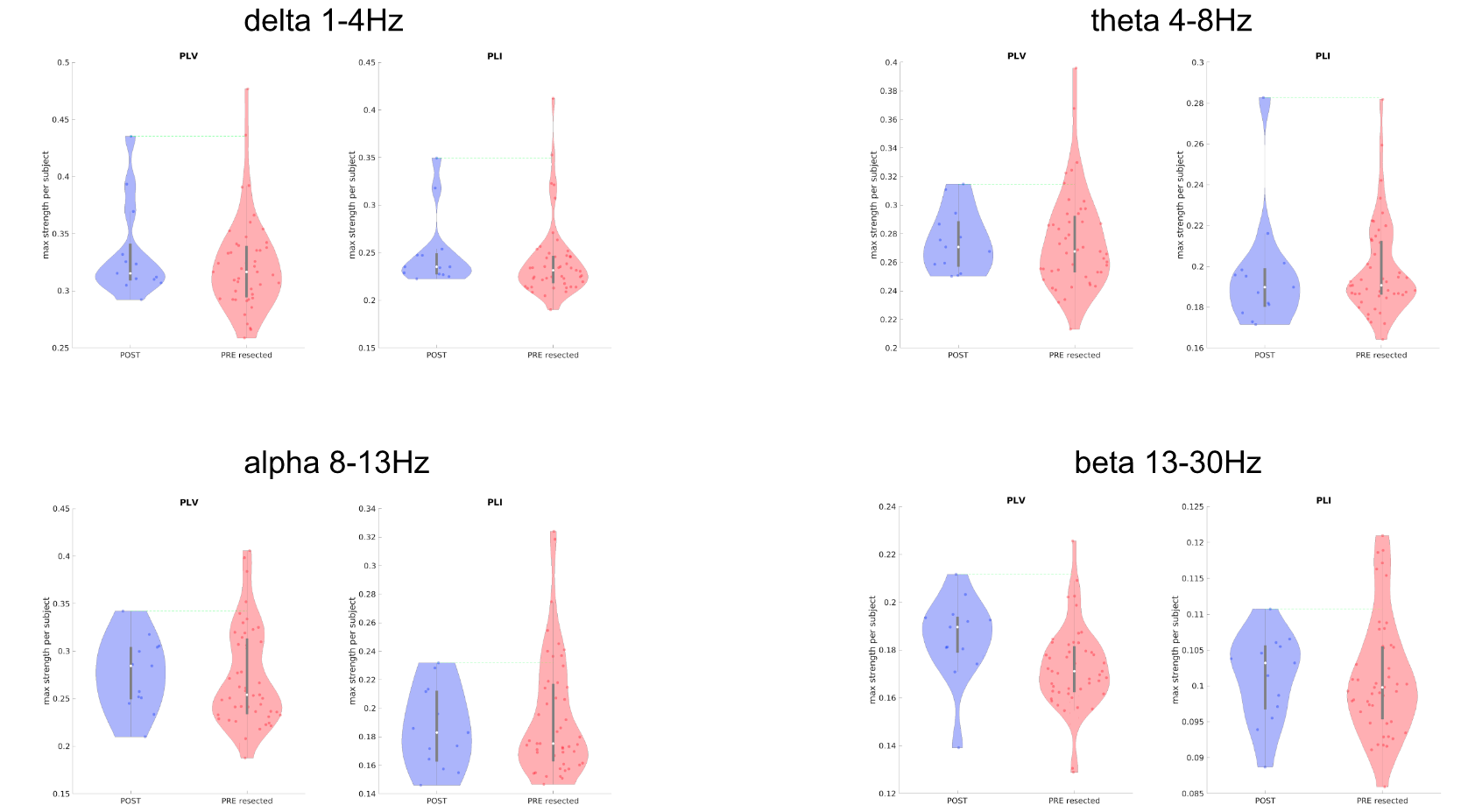


### Figure S2 Comparison between maximum biomarker values between pre-resection resected channels (red) in improved patients and post-resection channels in cured patients (blue) using a bipolar montage. Each dot represents the maximum value of the biomarker across all channels of each patient. The presence of an asterisk indicates that the two distributions are significantly different (p < 0.01 one-sided Kolmogorov-Smirnov test). Inside each violin-plot a boxplot is depicted in gray with the median value highlighted with a white dot. For each biomarker, the green line represents the threshold used to define the normal tissue (biomarker reference) using post-resection cured patients. PLV = Phase Locking Value; PLI = Phase Lag Index.

**Common average montage**

## *Measuring effect across all the channels*

### **
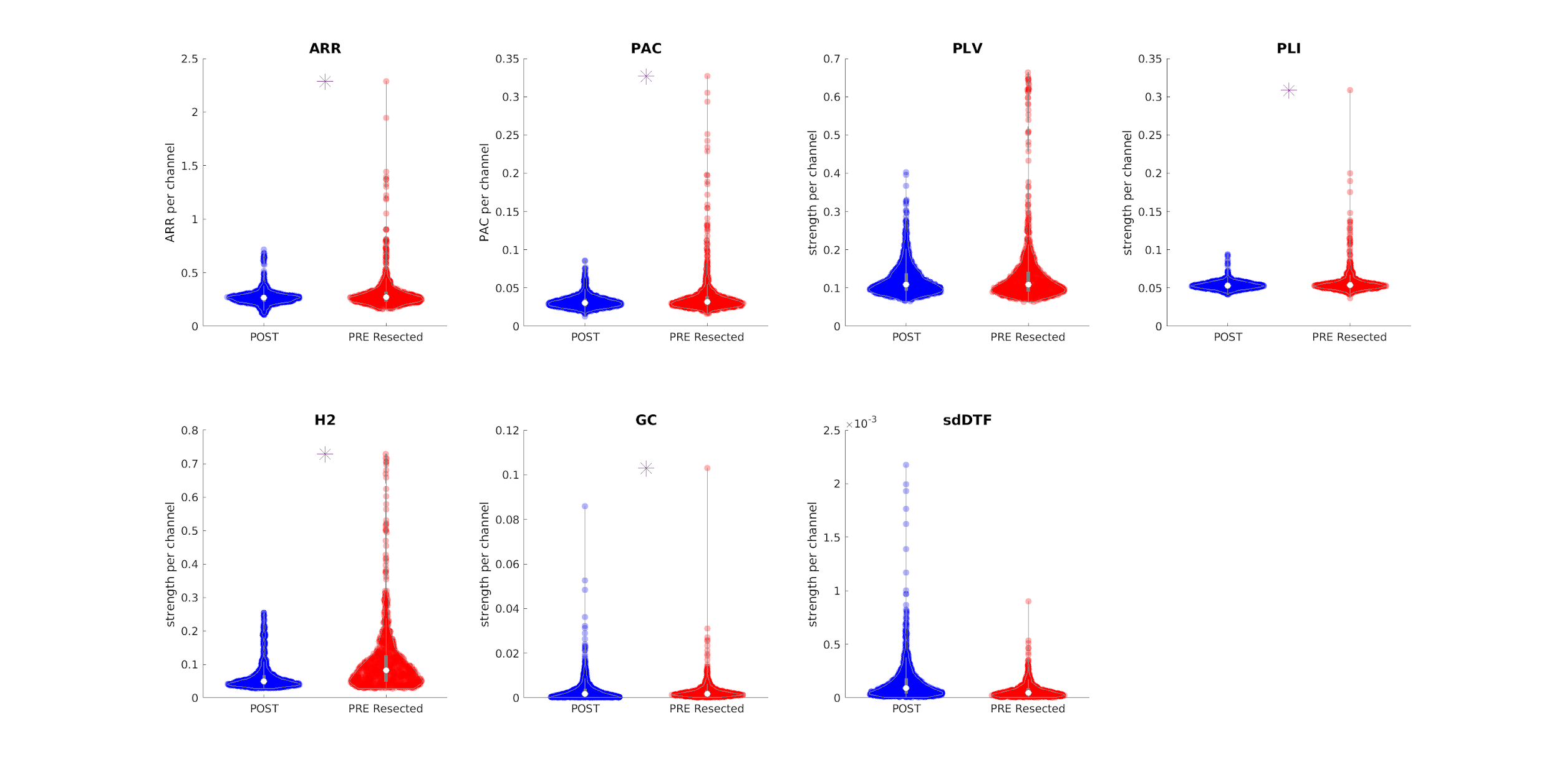
**Figure S3 Comparison between biomarker distributions values computed on pre-resection resected channels (red) and post-resection channels (blue) in cured patients using a common average montage. The presence of an asterisk indicates that the two distributions are significantly different (p < 0.01 one-sided Kolmogorov-Smirnov test). Note that each point for the univariate biomarkers (ARR and PAC) represents the value of the biomarker per channel, while the y-axis for the bi-/multi-variate biomarkers represents the strength. Inside each violin-plot a boxplot is depicted in gray with the median value highlighted with a white dot. ARR = Auto-regressive residual modulation; PAC = Phase Amplitude Coupling; PLV = Phase Locking Value; PLI = Phase Lag Index; H2 = non linear correlation coefficient; GC = Granger Causality; sdDTF = Short-time direct Directed Transfer Function.

## *Measuring effect using maximum per patient*

### **
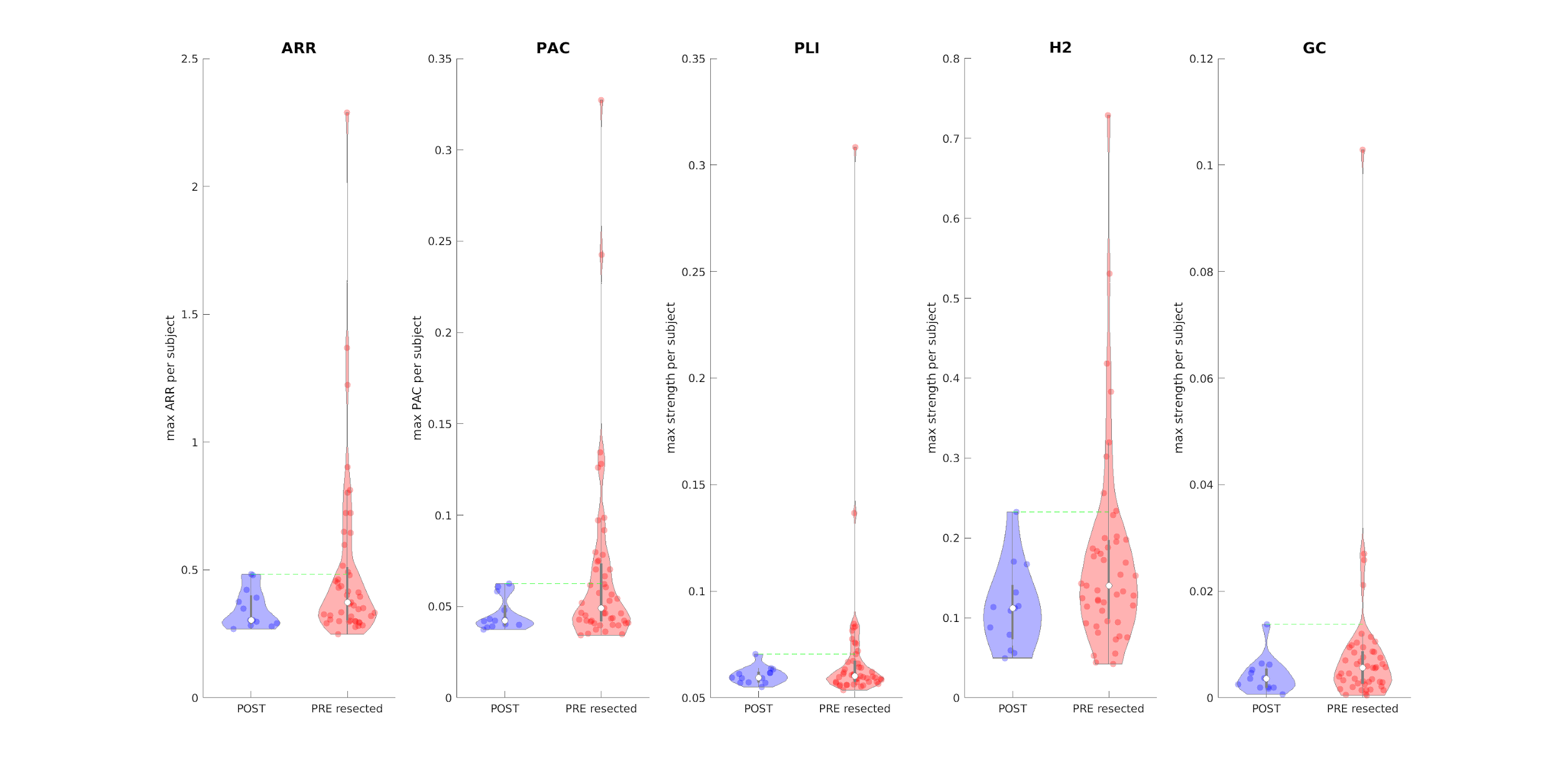
**Figure S4 Comparison between maximum biomarker values between pre-resection resected channels (red) in improved patients and post-resection channels in cured patients (blue) using a common average montage. Each dot represents the maximum value of the biomarker across all channels of each patient. The presence of an asterisk indicates that the two distributions are significantly different (p < 0.01 one-sided Kolmogorov-Smirnov test). Inside each violin-plot a boxplot is depicted in gray with the median value highlighted with a white dot. For each biomarker, the green line represents the threshold used to define the normal tissue (biomarker reference) using post-resection cured patients. ARR = Auto-regressive residual modulation; PAC = Phase Amplitude Coupling; PLI = Phase Lag Index; H2 = non linear correlation coefficient; GC = Granger Causality.

## *Pooling together all the biomarkers*

###

###
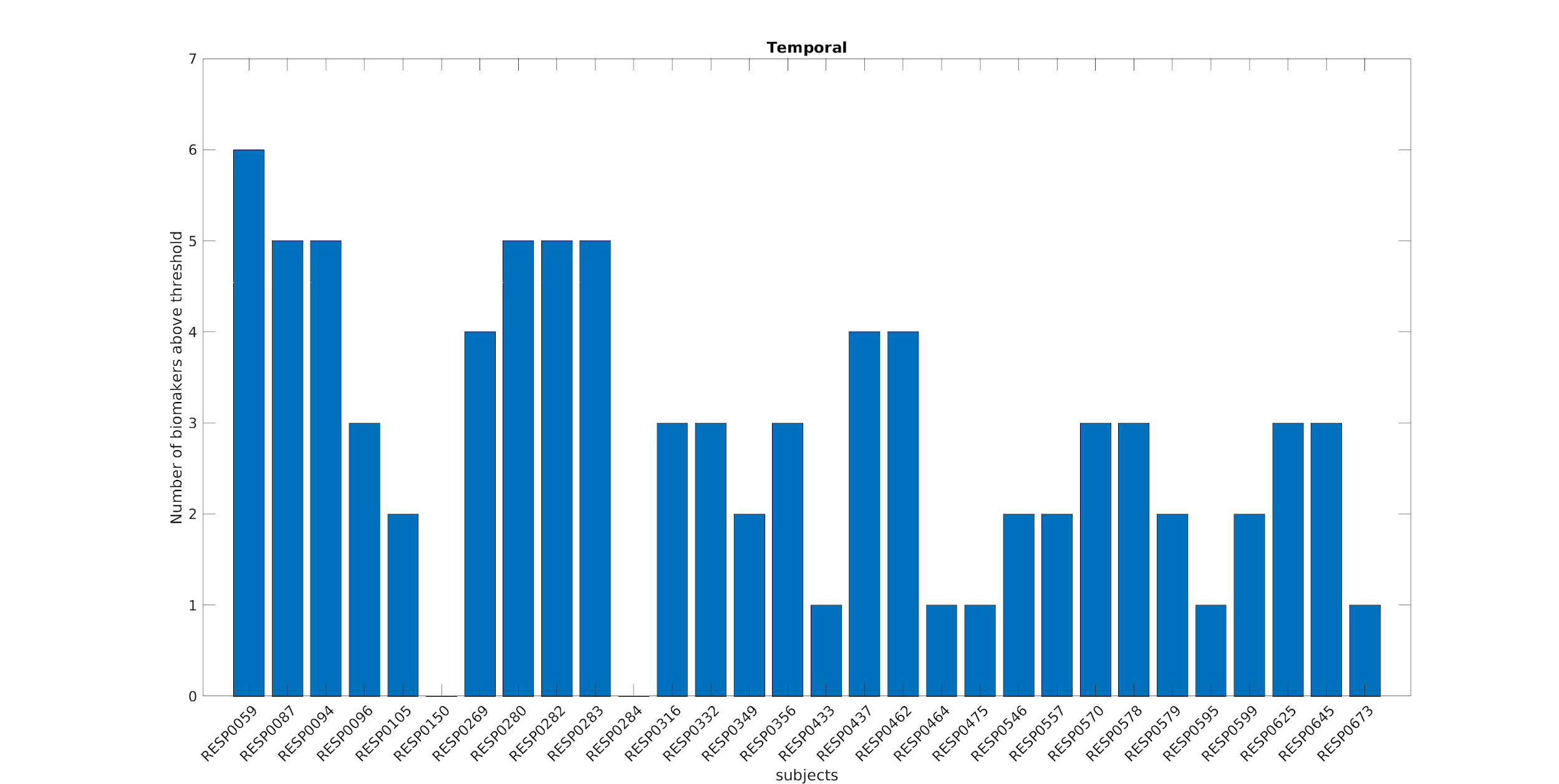
Figure S5 Number of biomarkers above the threshold for each temporal patient using a common average montage. On the x-axis each of the 30 improved temporal patients is displayed with a coded number. The y-axis represents the number of biomarkers above the specific threshold for each patient (computed independently for each biomarker and using only the temporal patients).

###
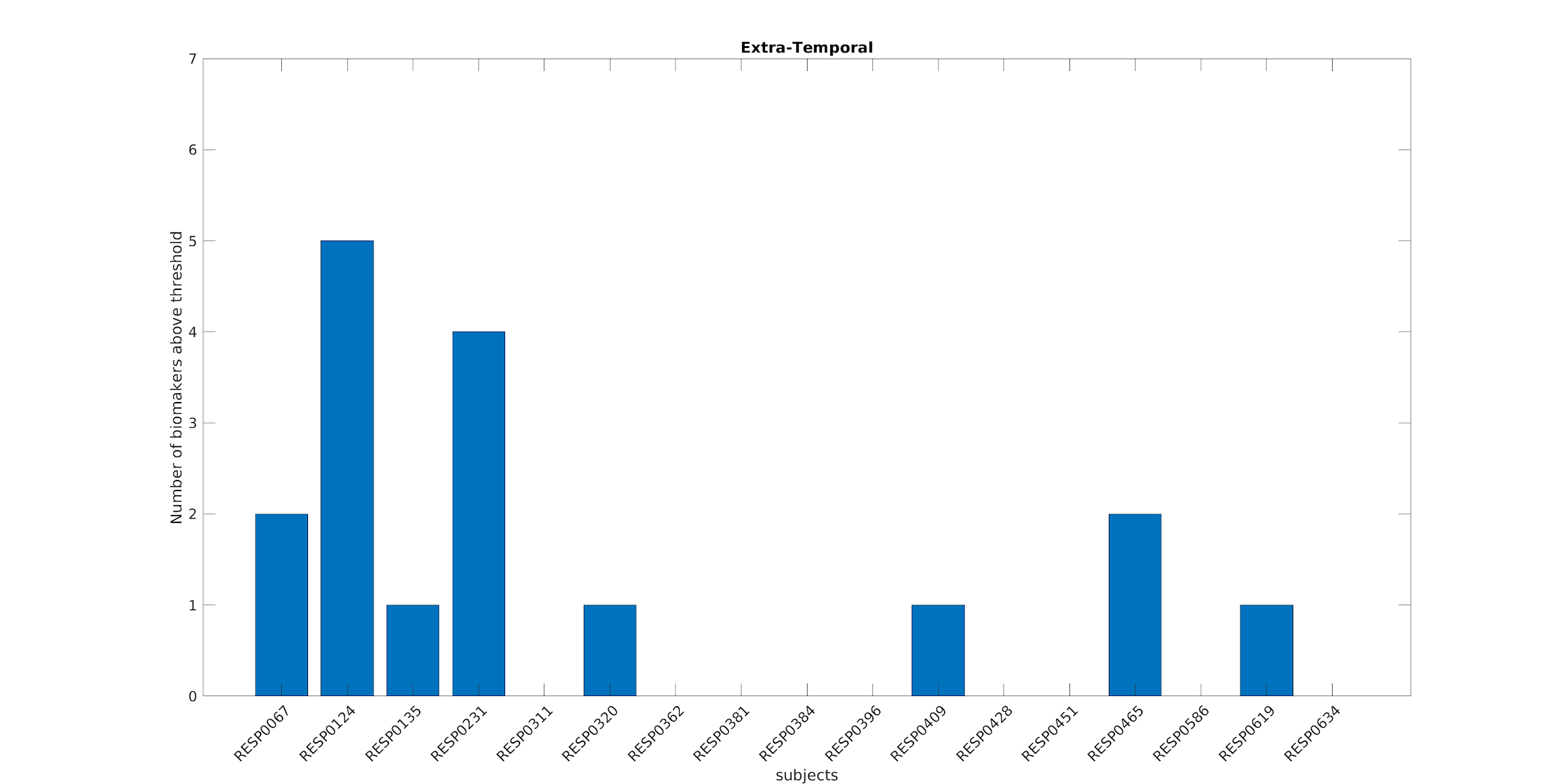
Figure S6 Number of biomarkers above the threshold for each extra-temporal patient using a common average montage. On the x-axis each of the 17 improved extra-temporal patients is displayed with a coded number. The y-axis represents the number of biomarkers above the specific threshold for each patient (computed independently for each biomarker and using only the extra-temporal patients).

**Bibliography**

1. [Niso, G. *et al.* What graph theory actually tells us about resting state interictal MEG epileptic activity. *Neuroimage Clin* **8**, 503–515 (2015).](http://paperpile.com/b/UFn3f7/SWCM)

2. [van Dellen, E. *et al.* MEG network differences between low- and high-grade glioma related to epilepsy and cognition. *PLoS One* **7**, e50122 (2012).](http://paperpile.com/b/UFn3f7/DXsn)

3. [Van Diessen, E. *et al.* Are high frequency oscillations associated with altered network topology in partial epilepsy? *Neuroimage* **82**, 564–573 (2013).](http://paperpile.com/b/UFn3f7/tOpx)
